# Giant fronto-ethmoidal osteoma – selection of an optimal surgical procedure^☆^

**DOI:** 10.1016/j.bjorl.2017.06.010

**Published:** 2017-07-17

**Authors:** Maria Humeniuk-Arasiewicz, Grażyna Stryjewska-Makuch, Małgorzata A. Janik, Bogdan Kolebacz

**Affiliations:** a Independent Public Research Hospital N° 7 of Silesian Medical University, Upper Silesian Medical Centre, Department of Laryngology and Laryngological Oncology, Katowice, Poland; b University of Silesia in Katowice, Institute of Computer Science, Department of Biomedical Computer Systems, Sosnowiec, Poland

**Keywords:** Osteoma, Surgery, Endoscopy, Ethmoid Sinus, Frontal Sinus, Osteoma, Cirurgia, Endoscopia, Seio etmoidal, Seio frontal

## Abstract

**Introduction:**

Osteomas of the paranasal sinuses are benign bone tumours that produce clinical signs depending on their size and location. In most reported cases large tumours are excised by an external approach or in conjunction with an endoscopic technique. Endoscopic treatment of such tumours is a huge challenge for the operator.

**Objective:**

Determine the optimal surgical approach by analysing giant osteomas of the frontal and ethmoidal sinuses in the literature.

**Methods:**

Group of 37 osteomas obtained from the literature review. A group of osteomas removed only by endoscopy was compared with a group in which an external approach (lateral rhynotomy or craniotomy) or combined external and endoscopic approach was applied.

**Results:**

The authors, based on the statistical analysis of the literature data, have found that the average size of osteomas excised endoscopically and those removed by external approaches does not differ statistically, when the osteomas are located in the ethmoidal cells (*p* = 0.2691) and the frontal sinuses (*p* = 0.5891).

**Conclusion:**

The choice of surgical method appears to be independent of the osteoma size and the decision is likely to be taken based on the experience of the surgeon, available equipment and knowledge of different surgical techniques.

## Resumo

**Introdução:**

Osteomas dos seios paranasais são tumores ósseos benignos cujas manifestações clínicas ocorrem em função de seu tamanho e localização. Na maioria dos casos relatados os tumores grandes são excisados por uma abordagem externa ou associada a uma técnica endoscópica. O tratamento endoscópico destes tumores ainda é um grande desafio para o cirurgião.

**Objetivo:**

Determinar a abordagem cirúrgica ideal ao analisar osteomas gigantes dos seios frontal e etmoidal na literatura.

**Método:**

Um total de 38 osteomas foram avaliados a partir da revisão da literatura. Um grupo de osteomas removidos apenas por cirurgia endoscópica foi comparado com um grupo para o qual foi utilizado uma abordagem externa (rinotomia lateral ou craniotomia) ou uma abordagem combinada, externa e endoscópica.

**Resultados:**

Os autores, com base na análise estatística dos dados da literatura, observaram que o tamanho médio dos osteomas excisados endoscopicamente e daqueles que foram removidos através de uma abordagem externa não diferiram estatisticamente, tanto para osteomas localizados no seio etmoidal (*p* = 0.2691) quanto para os localizados no seio frontal (*p* = 0.5891).

**Conclusão:**

A escolha do método cirúrgico parece ser independente do tamanho do osteoma e a decisão provavelmente será tomada com base na experiência prévia do cirurgião, nos equipamentos disponíveis e conhecimento de diferentes técnicas cirúrgicas.

## Introduction

Osteomas are frequent, benign osteogenic tumours of connective tissue arising from the proliferation of cancellous or cortical bone.^1^, ^2^ Craniofacial osteomas occur frequently, especially in the paranasal sinuses. Due to their slow asymptomatic growth, in most cases they are detected accidentally, in 3% of computed tomography (CT) scans and 1% of radiographs of the sinuses.^3^

Osteomas within the paranasal sinuses are most often localized in the fronto-ethmoidal region (95% of cases), involving the frontal sinus 60%–70%, and ethmoidal cells 20%–30%. Osteomas of the maxillary sinus occur in 5% of cases.^2^ Orbital involvement is the result of the spread of an osteoma from the neighbouring sinuses. Primary orbital involvement is exceedingly rare.^4^

The aetiology of osteomas still remains unclear. The role of inflammatory factors is suggested here as well as previous injuries and treatments within the nose and sinuses, or embryological theory.^1^, ^5^, ^6^ Genetic factors may affect the formation of osteomas in Gardner's syndrome with coexistence of intestinal polyps, epidermoid cysts and desmoid tumours.^7^

Janovic's observations suggest that patients with osteomas develop anatomical variations of the paranasal sinuses more frequently than patients without osteomas.^8^ There has been a higher incidence of osteomas in men than in women between the ages of 20–50 years in the ratio 2:1.^9^ The higher prevalence of osteomas in males may be due to more frequent exposure to injuries, and larger paranasal sinuses compared to women.^3^

Osteomas of the paransal sinuses of large sizes are, in most reported cases, excised by an external approach. Giant osteomas of the paranasal sinuses are tumours sized over 30 mm or weighing 110 g^10^ and they are usually excised by an external approach.^3^, ^4^, ^11^ Endoscopic treatment of such tumours is a major challenge for the operator. The authors of the study successfully removed osteomas sized below 30 mm located in the ethomoid sinuses by means of endoscopy, osteomas of the side portion of the frontal sinuses by craniotomy and tumours located in the area of the frontal fascia by endoscopy combined with the approach through the anterior wall of the frontal sinus. This paper presents the case of a fronto-ethmoidal giant osteoma successfully removed via endoscopy without intraoperative and postoperative complications.

The successful removal of the giant osteoma inspired the authors to perform a retrospective literature review and, based on the data, try to determine the optimal surgical approach to the giant osteoma of the ethmoidal cells invading the frontal recess or the orbit and located in the frontal sinuses.

## Case description

In 2016, a 75 year-old woman contacted the Department of Otolaryngology and Oncological Surgery complaining of epiphora of the right eye, persistent headaches, post-nasal drip, nasal obstruction, pain and pressure in the area of the right maxillary sinus. Several years before, the patient had been treated for chronic sinusitis.

**Figure 1:**
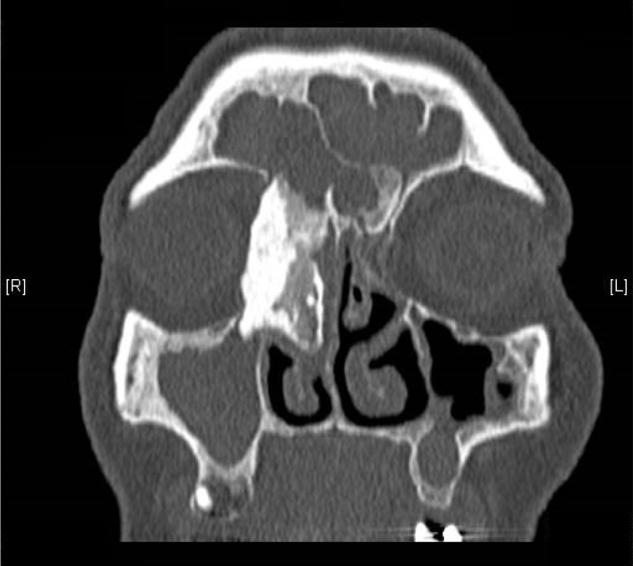
CT scan of the sinuses – fronto-ethmoidal osteoma sized about 39.5 × 19.8 × 19.4 mm, coronal plane.

**Figure 2:**
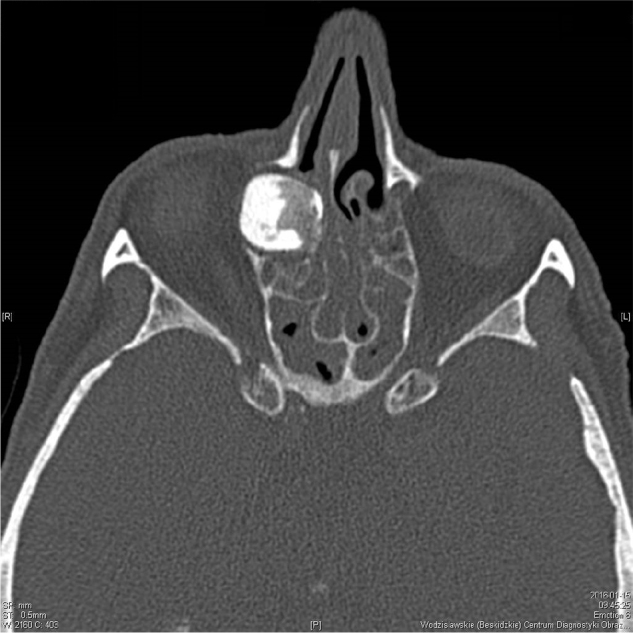
CT scan of the sinuses – fronto-ethmoidal osteoma, axial plane.

**Figure 3:**
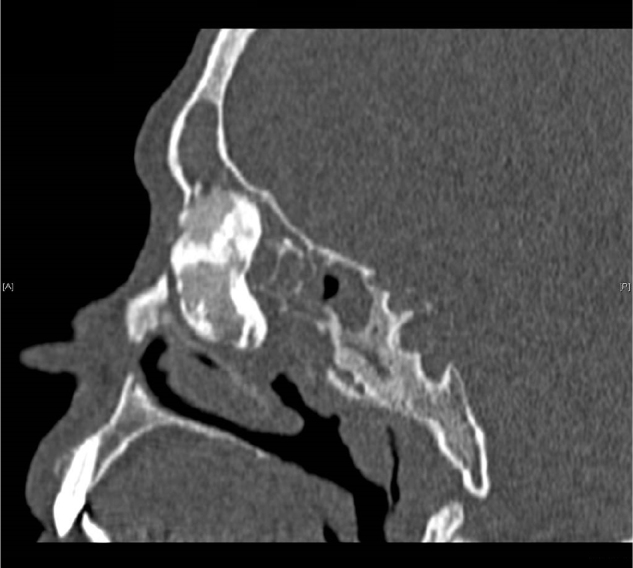
CT scan of the sinuses – giant fronto-ethmoidal osteoma, sagittal plane.

On the day of admission to hospital, a clinical trial showed asymmetry of the eyeballs with mild exophthalmos of the right eyeball, without impairment of its mobility and a hard swelling within the right angle of the orbit. There were no visual disturbances and neurological symptoms. The CT scan of the paranasal sinuses showed a nodular, calcified, polycyclic structure sized about 39.5 × 19.8 × 19.4 mm within the anterior ethmoidal cells on the right side, protruding into the right orbit, and putting pressure on the medial rectus muscle and the lacrimal sac, as well as invading the area of the frontal sinus and the right ostiomeatal complex. An osteoma with a diameter of about 9 mm was found in the ostium of the left frontal sinus. According to a radiologist, the shading of the right maxillary, ethmoidal, frontal and sphenoidal sinuses suggested massive mucosal hyperplasia and purulent secretions or mucus (Figure 1, Figure 2, Figure 3).

Under general anaesthesia and controlled hypotension, a solid bony structure was visualized endoscopically on the right side, filling the middle nasal meatus, ethmoidal cells and frontal recess. The middle turbinate got thinner, pressed by the tumour. Uncinectomy and antrostomy were performed on the right side. The medial portion of the tumour from the side of the middle turbinate, anterior ethmoidal cells and frontal recess was carefully removed using a drill. The remaining lesion was gently removed from the superior-medial portion of the maxillary sinus and the medial portion of the orbit. To enable the removal of the tumour through the nasal passage, the anterior segment of the inferior turbinate had to be removed. The material was sent for histopathological examination. The roof of ethmoidal cells, the remnants of posterior ethmoidal cells and the interior of the maxillary sinus were purified from polyps and purulent secretions. Then, uncinectomy and antrostomy were performed on the left side and the polyps were excised from the anterior and posterior ethmoidal cells using a microdebrider. No cerebrospinal fluid leak was observed during the surgery or in the postoperative period. The patient was discharged in good condition on the third day after the surgery.

An osteoma without evidence of malignancy was diagnosed in the histopathological examination of the tumour.

**Figure 4:**
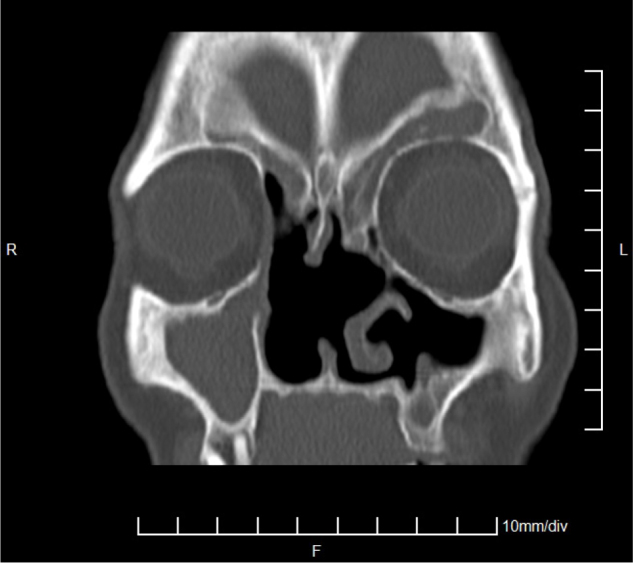
CT scan of the sinuses – 12 months after surgery, coronal plane.

**Figure 5:**
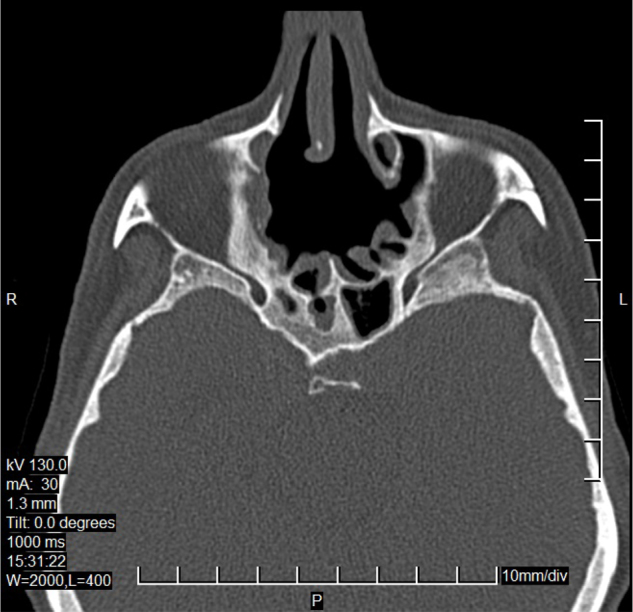
CT scan of the sinuses – 12 months after surgery, axial plane.

The patient has been under constant laryngological control for 14 months. An interview, clinical trial and imaging showed no recurrence of the tumour, and the symptoms reported by the patient before the surgery have completely resolved. Figure 4, Figure 5 present a CT scan of the sinuses performed in an operated patient 12 months after surgery.

Successful, endoscopic removal of the giant osteoma of the ethmoidal sinuses prompted the authors to analyze surgical procedures used to remove osteomas exceeding 30 mm in the greatest dimension and smaller ones. The selected publications describe osteomas located in the frontal or ethmoidal sinuses with the involvement of adjacent areas (frontal recess, orbit).

## Methods

The material, which was the basis of statistical analysis, was a group of 37 osteomas obtained from the literature review. From the available literature, only those works were selected for analysis in which the authors presented operated osteomas in the frontal plane in the sinus CT scan and it was possible to read the tumour dimensions. The rejected works included those in which the dimensions of osteomas were given in other projections in a CT scan or were calculated after tumour removal in a histopathological examination. A group of osteomas removed only by transnasal endoscopy was compared with a group in which an external approach (through the anterior wall of the frontal sinus, orbit, lateral rhynotomy or craniotomy), where an endoscope was not used in surgery, or combined external and endoscopic approach was applied. The external and combined method was for reasons of simplicity referred to as “other” in Table 1. The largest diameter of the tumour in the frontal plane in the CT scan of the paranasal sinuses was taken into consideration in the analysis. The size distribution of osteomas in each group was assessed by the Shapiro–Wilk test and graphically using histograms. The median was chosen as the measure of centre, and a quartile range as a measure of diversity. In order to compare the average size of osteomas removed endoscopically and with an external approach separately for the frontal sinuses and ethmoidal cells, an unpaired *t*-test was made or, in justified cases, its nonparametric equivalent, i.e. the Mann–Whitney *U* test. The statistical analysis was made using Statistica 12. A *p*-value less than 0.05 was considered as statistically significant.

**Table 1:** The group of osteomas obtained from the literature review.

Author	Localization	Method	Size (mm)
Authors of this paper	Ethmoid sinus	endoscopically	≥30

**Literature review**			
Mansour et al.^3^	Ethmoid sinus	other	≥30
Zouloumis et al.^4^	Ethmoid sinus	other	<30
Blanco Dominguez et al.^9^	Ethmoid sinus	Other	<30
Cheng et al.^10^	Ethmoid sinus	Other	≥30
Cheng et al.^10^	Frontal sinus	Other	≥30
Cheng et al.^10^	Frontal sinus	Other	≥30
Hazarika et al.^11^	Frontal sinus	Other	≥30
Zhuang et al.^12^	Ethmoid sinus	Other	≥30
Oleś et al.^13^	Frontal sinus	Endoscopically	≥30
Oleś et al.^13^	Frontal sinus	Endoscopically	≥30
Stręk et al.^14^	Ethmoid sinus	Endoscopically	<30
Stręk et al.^14^	Ethmoid sinus	Endoscopically	<30
Stręk et al.^14^	Ethmoid sinus	Endoscopically	<30
Stręk et al.^14^	Frontal sinus	Endoscopically	<30
Panagiotopoulos et al.^15^	Frontal sinus	Other	≥30
Nagashima et al.^16^	Frontal sinus	Other	<30
Savastano et al.^17^	Frontal sinus	Other	≥30
Beitzke et al.^18^	Frontal sinus	Other	≥30
Kamide et al.^19^	Ethmoid sinus	Other	<30
Kim^20^	Ethmoid sinus	Endoscopically	<30
Torun et al.^21^	Ethmoid sinus	Endoscopically	≥30
Simsek et al.^22^	Ethmoid sinus	Endoscopically	≥30
Li et al.^23^	Ethmoid sinus	Endoscopically	<30
Naraghi et al.^24^	Ethmoid sinus	Endoscopically	≥30
Akmansu et al.^25^	Ethmoid sinus	Endoscopically	≥30
Saetti et al.^26^	Ethmoid sinus	Endoscopically	≥30
Kim^27^	Frontal sinus	Endoscopically	<30
Gerbrandy et al.^28^	Ethmoid sinus	Other	≥30
Maharjan et al.^29^	Ethmoid sinus	Other	≥30
Lodha et al.^30^	Ethmoid sinus	Other	<30
Ansari et al.^31^	Ethmoid sinus	Other	≥30
Müslüman et al.^32^	Frontal sinus	Other	≥30
Müslüman et al.^32^	Ethmoid sinus	Other	≥30
Manaka et al.^33^	Frontal sinus	Other	≥30
Karbassi et al.^34^	Ethmoid sinus	Other	≥30
Saati et al.^35^	Ethmoid sinus	Other	≥30
Alotaibi et al.^36^	Ethmoid sinus	Endoscopically	≥30

## Results

Among the collected literature data, including the case described by the authors, 31.58% of all osteomas were localized in the ethmoidal cells and excised endoscopically, a similar amount (34.21%) constituted osteomas located in the ethmoidal cells and removed by an external approach. The other cases were located in the frontal sinuses, of which only 1/3 were removed endoscopically. Among osteomas excised endoscopically, 75% were localized in the ethmoidal cells, the remaining 25% in the frontal sinuses. In the case of methods using an external approach, 59.1% of cases were located in the ethmoidal cells, the remaining 40.9% in the frontal sinuses. In the case of osteomas located in the ethmoidal cells, 48% were excised endoscopically and 52% by an external approach. In none of the cases the differences in numbers were statistically significant, so it is not possible to conclude that the choice of methods is associated with the location of osteomas.

Among osteomas located in the ethmoidal cells, 64% were giant osteomas (≥30 mm): 43.75% of them were removed endoscopically, and the remaining 56.25% were removed by an external approach. Osteomas of the ethomoid cells sized less than 30 mm accounted for 36%, of which 55.56% were excised endoscopically, the remaining 44.44% by an external approach. Of all osteomas located in the ethmoidal cells, 28% were giant osteomas excised endoscopically, and 36% giant osteomas excised by an external approach. As in the case of the location of osteomas and the chosen method, there was no relationship between the size of the osteoma of the ethmoidal cells and the method of its removal. The same relationships are in the case of osteomas located in the frontal sinuses, but of all giant osteomas (representing 73.92% of all cases located in the frontal sinuses); only 20% were removed endoscopically.

## Discussion

Osteomas belong to benign paranasal sinuses that may be the cause of various clinical symptoms depending on the location, size and direction of tumour growth. Some tumours are accidentally diagnosed during radiological examinations performed for other reasons. Computed tomography, where osteomas typically occur as a thick, sclerotic, homogeneous, well-defined structure, is sufficient to diagnose and accurately plan the surgical approach.^31^ Four pathological types of osteomas have been described: 1) ivory-hard, dense, mature bone with total absence of Haversian canals, 2) compact lamellar structure with small Haversian canals, 3) spongiose-periphery of compact bone with radial septa and intervening marrow spaces, 4) mixed-bone and fibrous tissue.^37^ Types 3 and 4 are more rapidly growing.

Osteomas are characterized by slow asymptomatic growth that can take years until the first symptoms of the disease appear. Most commonly they include headaches, facial deformities, vertigo, sinusitis, disorders of nasal obstruction.^9^ The symptoms of the disease appear when normal sinus drainage becomes impaired due to the obstruction of its ostium by the tumour growth. Ocular and central nervous system symptoms result from the spread of osteomas located in the fronto-ethmoid region and can cause exophthalmos, dacryorrhea, retrobulbar pain, double vision.^2^, ^4^, ^9^, ^36^ In some cases they may cause intracranial complications such as cerebral abscess, meningitis, mucocele, and even lead to cerebral oedema as in the case of the ethomoid osteoma described by Kamid, manifesting itself as headaches and mild hemiparesis.^15^, ^16^, ^18^, ^19^, ^38^ Giant ethomoid osteomas are rare, accounting for approximately 0.9%–5.1% of all orbital tumours.^31^ Ethomoid osteomas can produce symptoms much faster than those located in the frontal sinuses due to limited space in the ethomoid region and, consequently, due to faster invasion of neighbouring structures.^34^

Indications for surgical treatment of osteomas are ambiguous. Many authors suggest constant observation of asymptomatic tumours or those discovered accidently by performing CT scans regularly every 12 months.^14^ Magnetic resonance imaging is useful in differential diagnosis and in cases of orbital involvement and intracranial spread.^7^

Surgery is recommended in cases of significant tumour growth^10^ accompanied by the appearance of clinical symptoms, involvement of the orbit or anterior cranial fossa and the resulting complications.^9^, ^16^, ^17^, ^36^ In the case of the asymptomatic osteoma of the frontal sinus described by Hazarika, the patient had been under constant observation for 10 years, the tumour sized 38.1 mm was operated, and then the patient developed symptoms such as headaches and excessive epiphora.^11^ Panagiotopoulos proposes surgical removal of small osteomas before tumour progression, the appearance of symptoms and intracranial complications.^15^ It is suggested that osteomas involving the area of the nasolacrimal duct or more than half of the frontal sinus should be excised.^3^ Each sphenoid sinus osteoma requires rapid surgical treatment regardless of symptomatology, due to the possibility of optic neuropathy resulting from oppression of a slow-growing tumour and blindness.^7^, ^9^ Lee describes the case of sphenoid sinus osteoma, which was observed in accordance with the “wait-and-see policy”. Surgical treatment was not initiated because of the small size of the lesion.^39^ In recent years, with the development of endoscopic sinus surgery, the removal of paranasal sinus osteomas via endoscopy has become a method of choice because of the low morbidity rate, aesthetic aspects, lower cost of treatment and greater experience of surgeons.

Literature analysis conducted by the authors showed that among osteomas excised endoscopically, 75% were localized in the ethmoid cells, the remaining 25% in the frontal sinuses. The results suggest that in the case of osteomas below 30 mm located in the ethomoid sinuses, endoscopy may be the surgical method of choice. Giant osteomas in the ethomoid sinuses were removed via endoscopy and the external approach equally often. Lee claims that the endoscopic approach allows for the removal of all ethmoid osteomas with skull base or lamina papyracea involvement.^39^ Alotaibi describes the case of an osteoma of the anterior ethmoidal cells sized 30 × 25 × 15 mm growing in the direction of the orbit, which was excised endoscopically using neuronavigation, as in the case of the tumour sized 30 × 20 × 15 mm described by Zhuang.^12^, ^36^ Endoscopic excision of osteomas from the ethomoid cells is recommended, but insufficient when the lesions spread outside the sinuses.^40^ Karbassi draws attention to the cases, described in the literature, of giant osteomas in the ethomoid region removed endoscopically, which were not accompanied by orbital complications. He suggests that external surgery is the proper method for removing osteomas with deep invasion of the orbit.^34^

The results of statistical analysis of the available literature showed that there was no relationship between the size of the osteoma of the ethmoidal cells and the method of its removal. The case of giant osteoma described in this paper confirms the validity of endoscopic treatment. The analysis was different in the case of the frontal sinuses. All giant osteomas (representing 73.92% of all cases located in the frontal sinuses), only 20% were removed endoscopically. Chiu recommended endoscopic resection of small frontal osteomas medial to the sagittal line passing through the lamina papyracea (Grade I and II), while using the external approach if the location was lateral to the sagittal line passing through the lamina papyracea or filling the frontal sinus (Grade III and IV).^41^ Savastano suggests removal of osteomas of the frontal sinuses with an external approach if they are located on the front or side wall of the sinus. According to Hazarika, osteomas of the frontal sinuses arising from the back wall of the sinus with a wide base should not be operated endoscopically due to complications, including cerebrospinal fluid leak.^11^, ^17^ In the literature, there are cases of endoscopic treatment of frontal sinus osteomas of a diameter greater than 3 cm using Draf III access.^13^ Endoscopic treatment of lesions in the frontal sinuses is possible in cases of tumours located near the frontal recess.^17^ Endoscopic surgery of osteomas in the frontal recess region may affect the postoperative course. Based on operational experience, Lodha describes the possibility of secondary sinusitis after endoscopic drilling, which can cause osteitis with frontal recess stenosis.^42^ Nagashima describes the use of craniotomy in the case of osteomas with involvement of the orbit, anterior cranial fossa, or a portion of the frontal sinus.^16^

According to Müslüman, multicompartmental osteomas should be excised via the external approach due to the possibility of recurrence, as in the case of the described giant fronto-ethomoid osteoma with orbital involvement, using the transcranial approach for radical excision of the lesion. In the case of giant osteomas, the transcranial surgical approach is safer and more effective compared to other external and endoscopic operations used as the only surgical method.^32^ According to Karapantzos, the transcutaneous paranasal approach (rhynotomy) allows, to a greater extent, for proper diagnosis and protection of the lacrimal drainage apparatus.^43^

Verma describes an attempt to remove endoscopically a maxilliary sinus tumour sized 4 × 5 × 3 cm, which ended with the Weber-Fergusson approach due to the difficult access to the back and lateral part of the tumour.^2^ Maxillary osteomas located in the upper part of the maxillary sinus can be treated via the endoscopic approach.^39^

Recurrence of osteomas after surgery is very rare, about 10%, usually after earlier incomplete removal of the tumour.^15^, ^44^ There is a possibility of partial osteoma removal as long as its average growth rate is not more than 1.61 mm/year.^19^, ^45^ Then the constant observation of patients and regular imaging tests are necessary in order to exclude recurrence. It is often sufficient to perform a radiogram every 6 months for several years after surgery.^14^

## Conclusion

Based on the statistical analysis of literature data and having regard to the described case, the authors have found that the average size of osteomas excised endoscopically and those removed by the external approach does not differ statistically, in the case of osteomas located both in the ethmoidal cells (*p* = 0.2691) and the frontal sinuses (*p* = 0.5891).

The choice of surgical method appears to be independent of the osteoma size and the decision is likely to be taken based on the past experience of the surgeon, available equipment and knowledge of different surgical techniques within the frontal sinuses. It appears that the endoscopic approach, being less traumatic, leaving no scars and allowing shorter stay in hospital, will be the method of choice especially in fronto-ethmoidal cases.

## Conflicts of interest

The authors declare no conflicts of interest.
